# Intravenous dantrolene in hypermetabolic syndromes: a survey of the U.S. Veterans Health Administration database

**DOI:** 10.1186/s12871-022-01841-z

**Published:** 2022-09-19

**Authors:** Stanley N. Caroff, Christopher B. Roberts, Henry Rosenberg, Joseph R. Tobin, Stacey Watt, Darlene Mashman, Sheila Riazi, Rosalind M. Berkowitz

**Affiliations:** 1 Department of Psychiatry, Corporal Michael J. Crescenz VA Medical Center and the University of Pennsylvania Perelman School of Medicine, Philadelphia, PA 19104 USA; 2 Center for Health Equity Research and Promotion, Corporal Michael J. Crescenz VA Medical Center, Philadelphia, PA USA; 3 Malignant Hyperthermia Association of the United States, Sherburne, NY USA; 4 Wake Forest University, Winston Salem, NC USA; 5 University of Buffalo, Buffalo, NY USA; 6 Emory University School of Medicine, Children’s Healthcare of Atlanta, Atlanta, GA USA; 7 University of Toronto, Toronto, ON Canada

**Keywords:** Dantrolene, Malignant hyperthermia, Neuroleptic malignant syndrome, Serotonin syndrome, Heatstroke, Sepsis, Rhabdomyolysis, Anesthetic drugs, Antipsychotic drugs, Succinylcholine

## Abstract

**Background:**

Intravenous dantrolene is often prescribed for hypermetabolic syndromes other than the approved indication of malignant hyperthermia (MH). To clarify the extent of and indications for dantrolene use in conditions other than MH, we sought to document current practices in the frequency, diagnoses, clinical characteristics and outcomes associated with dantrolene treatment in critical care settings.

**Methods:**

Inpatients receiving intravenous dantrolene from October 1, 2004 to September 30, 2014 were identified retrospectively in the U.S. Veterans Health Administration national database. Extracted data included; diagnoses of hypermetabolic syndromes; triggering drugs; dantrolene dosages; demographics; vital signs; laboratory values; in-hospital mortality; complications; and lengths of stay. Frequency and mortality of patients who did not receive dantrolene were obtained in selected diagnoses for exploratory comparisons.

**Results:**

Dantrolene was administered to 304 inpatients. The most frequent diagnoses associated with dantrolene treatment were neuroleptic malignant syndrome (NMS; *N* = 108, 35.53%) and sepsis (*N* = 47, 15.46%), with MH accounting for only 13 (4.28%) cases. Over half the patients had psychiatric comorbidities and received psychotropic drugs before dantrolene treatment. Common clinical findings in patients receiving dantrolene included elevated temperature (mean ± SD; 38.7 ± 1.3 °C), pulse (116.33 ± 22.80/bpm), respirations (27.75 ± 9.58/min), creatine kinase levels (2,859.37 ± 6,646.88 IU/L) and low pO_2_ (74.93 ± 40.16 mmHg). Respiratory, renal or cardiac failure were common complications. Mortality rates in-hospital were 24.01% overall, 7.69% in MH, 20.37% in NMS and 42.55% in sepsis, compared with mortality rates in larger and possibly less severe groups of unmatched patients with MH (5.26%), NMS (6.66%), or sepsis (41.91%) who did not receive dantrolene.

**Conclusions:**

In over 95% of cases, dantrolene administration was associated with diagnoses other than MH in critically-ill patients with hypermetabolic symptoms and medical and psychiatric comorbidities. Exploratory survey data suggested that the efficacy and safety of dantrolene in preventing mortality in hypermetabolic syndromes other than MH remain uncertain. However, randomized and controlled studies using standardized criteria between groups matched for severity are essential to guide practice in using dantrolene.

**Supplementary Information:**

The online version contains supplementary material available at 10.1186/s12871-022-01841-z.

## Background

Dantrolene, initially developed as an oral drug for spasticity, dissociates excitation–contraction coupling in skeletal muscle by binding to the ryanodine-receptor-1 (RyR1) and inhibiting calcium release from the sarcoplasmic reticulum [1–3]. Intravenous dantrolene received approval from the U.S. Food and Drug Administration for treating malignant hyperthermia (MH) during general anesthesia, reducing mortality from 70–80% to less than 10% [2–6].

MH is a pharmacogenetic disorder of skeletal muscle presenting as a hypermetabolic response to volatile anesthetics or succinylcholine. Classic signs include hyperthermia, tachycardia, tachypnea, increased carbon dioxide production and oxygen consumption, acidosis, hyperkalemia, muscle rigidity, and rhabdomyolysis. Estimates of the incidence of MH episodes range from 1:5,000 to 1:100,000 general anesthetics [3, 7]. However, the true incidence of MH episodes in hospital is uncertain as the accuracy of discharge records which have been used to identify MH cases has been questioned [8]. For example, in a recent report of hospital billing records in which patients with a discharge diagnosis of MH were identified, only 23.4% had a likely MH episode while 23.4% had hyperthermia attributed to other causes when their medical records were reviewed by an expert panel of anesthesiologists [9].

MH is inherited in an autosomal dominant pattern with susceptibility linked primarily to the RYR1 gene [4]. The prevalence of RYR1 genetic variants associated with MH-susceptibility may be as high as 1:400 to 1:8500 among individuals in the general population [3, 10], which suggests a possible broader risk for hypermetabolic reactions outside of anesthetic settings [11–17]. In fact, there are a number of related life-threatening syndromes with MH-like hypermetabolic symptoms including hyperthermia, muscle rigidity, rhabdomyolysis, and autonomic lability which have been reported in other clinical settings. For example, recent studies suggest a genetic relationship between MH-susceptibility and exertional heatstroke or exertional rhabdomyolysis, which provided a rationale for studies of the possible efficacy of dantrolene in heat-related illness with mixed results to date [18–22]. Alternative phenotypic manifestations of RYR1 variants have been speculated as predisposing to neuroleptic malignant syndrome (NMS) [23, 24], serotonin syndrome [25], stimulant abuse [26, 27], parkinsonism-hyperpyrexia syndrome [28], sepsis [29], toxicity of uncoupling agents dinitrophenol and aspirin [30], and others [31–33]. Because of the clinical parallels with MH, dantrolene has been used “off-label” (i.e., for disorders other than the officially approved indication) for these syndromes with some positive results [26, 27, 32, 34–42].

Given limited data on current practices and the likelihood that anesthesiologists and other clinicians will encounter these hypermetabolic syndromes, our primary objective was to document current practices in relation to the frequency, diagnoses, clinical characteristics and outcomes of intravenous dantrolene treatment. Our primary hypothesis was that dantrolene is prescribed most often for diagnoses other than MH, and secondarily, that a common profile of targeted hypermetabolic symptoms could be identified across diagnostic categories that prompted dantrolene administration. Better understanding of the effectiveness of dantrolene in hypermetabolic syndromes after exposure to environmental, behavioral or toxic challenges could inform evidence-based practice guidelines.

## Methods

In a retrospective descriptive study, data were drawn from the U.S. Veterans Health Administration (VA) national database, which included healthcare records on 5.9 million veterans. Extracted data included demographic and clinical characteristics of veterans, hospitalizations, pharmacy fills and diagnoses (International Classification of Diseases, Ninth Revision, Clinical Modification [ICD-9-CM]; Supplementary Table 1) [43]. Veterans were included if they received intravenous dantrolene during an inpatient admission in any VA facility nationwide from October 1, 2004 to September 30, 2014. Apart from requiring dantrolene administration, there were no other inclusion or exclusion criteria. Only the last admission was counted if dantrolene was administered on more than one admission. The institutional review board at the Corporal Michael J. Crescenz VA Medical Center approved this project as an exempted retrospective study and determined that informed consent was not required.

Dantrolene administration was characterized by years, dose, days, and intervals of administration. The bed service at the time of admission and in which dantrolene was administered were recorded. Individuals were characterized by age, sex, race/ethnicity, and geographic district. Co-morbidities with psychiatric or substance use disorders were noted. Records were screened using ICD-9-CM codes for 12 diagnoses associated with hypermetabolic syndromes as diagnosed by clinicians and listed as primary or secondary diagnoses on admission or discharge during the index hospitalization in which dantrolene was administered; MH, heatstroke, myopathies (e.g., central core disease), diabetes with hyperosmolarity, NMS, Parkinson’s disease, serotonin syndrome, rhabdomyolysis, masseter muscle rigidity (a *forme fruste *of MH), severe sepsis, stimulant abuse, and fever of unknown origin. In a subgroup inspection to examine the reliability of the diagnosis in patients who were coded in the database as having MH, we used the Malignant Hyperthermia Clinical Grading Scale to review individual medical records and rate the likelihood of MH [44]. This is a standardized rating scale based on expert consensus that uses the sum of raw score points assigned to a list of symptom indicators present in the patient, e.g., rigidity, temperature, etc., that is then used to rank the likelihood of the diagnosis of MH ranging from a raw sum score of zero, interpreted as “almost never” MH to a score of ≥ 50 interpreted as “almost certain” to be MH.

Data on length of stay and mortality during hospitalization were extracted. Complications arising during hospitalization (calculated by the number of diagnoses at discharge minus the number present on admission) included; acute renal failure, rhabdomyolysis, disseminated intravascular coagulation, pulmonary embolus, cardiac arrest, congestive heart failure, respiratory failure, phlebitis, and compartment syndrome.

Possible drugs triggering hypermetabolic syndromes were defined by prescriptions starting from 10 days before up to and including the first date when dantrolene was started, including; volatile anesthetics, succinylcholine, antipsychotics, antidepressants, monoamine oxidase inhibitors, antiparkinsonian drugs, and antimigraine triptans. Vital signs associated with hypermetabolic syndromes were defined as the highest value (except for the lowest value for pulse oximetry) recorded from 10 days before up to and including the first date when dantrolene was prescribed. Laboratory values were defined as the highest values recorded (except for lowest values for partial pressure of oxygen [pO_2_] and pH) from 10 days before up to and including the first date when dantrolene was prescribed.

For secondary exploratory purposes only, the frequency and mortality of patients who *did not* receive dantrolene were also determined for selected diagnoses; MH, chosen as the only approved indication for dantrolene; NMS and severe sepsis, chosen as representative of syndromes that are not approved for treatment with dantrolene; and heatstroke, chosen because of evidence suggesting genetic overlap with MH as a phenotypic manifestation of RYR1 mutations.

Continuous data are presented descriptively as means ± standard deviations (SD) plus ranges and percentiles of values, while categorical data are presented as percentages. Due to the non-randomized, uncontrolled and unmatched nature of the data that was extracted for exploratory descriptive comparisons, no statistical comparisons were made between patients who received dantrolene and those who did not.

## Results

Intravenous dantrolene was administered nationwide in 304 patients over the 10-year study period. A mean ± SD of 31.11 ± 6.30 cases (range 23–44) were reported each year. The two diagnoses most often associated with dantrolene administration were NMS (*N* = 108, 35.53%) and severe sepsis (*N* = 47, 15.46%) (Table 1). In over 95% of cases, dantrolene was administered off-label for unapproved conditions as only 13 (4.28%) were diagnosed with MH. When considering all patients in the database, dantrolene was administered only to a minority of patients with these three selected diagnoses; MH (*N* = 13/32; 40.63%), NMS (*N* = 108/754; 14.32%), or severe sepsis (*N* = 47/53,097; 0.08%). In 24 (7.90%) of the 304 cases who received dantrolene, it was prescribed for fevers of unknown origin. Finally, 33 (10.86%) of the 304 cases received dantrolene for unclear reasons without connection to any of the pre-selected ICD-9-CM diagnoses that have been associated with MH-like syndromes.

**Table 1:** Twelve selected diagnoses associated with dantrolene treatment during index hospitalizations (*N* = 304)

Primary/secondary diagnoses	N	(%)
**Neuroleptic Malignant Syndrome**	**108**	**(35.53)**
**Severe Sepsis**	**47**	**(15.46)**
**Rhabdomyolysis**	**45**	**(14.80)**
**Fever of Unknown Origin**	**24**	**(7.90)**
**Parkinson’s Disease**	**22**	**(7.24)**
**Malignant Hyperthermia**	**13**	**(4.28)**
**Serotonin Syndrome**	**10**	**(3.29)**
**Masseter Muscle Rigidity**	**1**	**(0.33)**
**Stimulant Use**	**1**	**(0.33)**
**Heatstroke**	**0**	**(0.00)**
**Myopathies**	**0**	**(0.00)**
**Diabetes with Hyperosmolarity**	**0**	**(0.00)**

Demographic data and clinical characteristics revealed that patients who received dantrolene were predominately older males, three quarters of whom were white and one fifth African American (Table 2). Psychiatric (*N* = 149; 49.01%) and substance use (*N* = 80; 26.32%) disorders were frequent co-morbidities. Use of dantrolene varied by geographic district from Pacific-based facilities (*N* = 35; 11.51%) to the Midwest and Continental districts (combined *N* = 154; 50.66%). Fourteen patients had received dantrolene on previous admissions. Patients who received dantrolene during their hospital stay were initially admitted to a medical (*N* = 206; 67.76%), psychiatric (*N* = 49; 16.12%), or surgical (*N* = 38; 12.50%) service, but received dantrolene primarily on medical (*N* = 261; 85.86%) and surgical (*N* = 43; 14.14%) services.

**Table 2:** Demographics of VA inpatients receiving intravenous dantrolene (*N* = 304)

Characteristic	N	(%)
**Age (mean ± SD)**	**60.77 ± 12.49**	
**Gender**		
**Female**	**11**	**(3.62)**
**Male**	**293**	**(96.38)**
**Race**		
**American Indian/Alaska Native**	**2**	**(0.66)**
**Asian**	**1**	**(0.33)**
**African American**	**65**	**(21.38)**
**Hispanic**	**4**	**(1.32)**
**White**	**230**	**(75.66)**
**Unknown**	**2**	**(0.66)**
**Body Mass Index (mean ± SD)**	**28.75 ± 7.00**	
**Co-Morbidities**		
**Psychiatric**	**149**	**(49.01)**
**Substance Use**	**80**	**(26.32)**
**District**		
**North Atlantic**	**52**	**(17.11)**
**Southeast**	**63**	**(20.72)**
**Midwest**	**77**	**(25.33)**
**Continental**	**77**	**(25.33)**
**Pacific**	**35**	**(11.51)**

*VA* Veterans Health Administration, *SD* Standard deviation

In terms of pharmacological variables, the mean total dose of dantrolene prescribed per patient during index hospitalizations was 765.36 mg, administered over 2.66 ± 3.07 days. The mean dose per day for a 70 kg patient calculates to 4.11 mg/kg/day, within the recommended dosing guidelines of dantrolene for MH [7, 45]. If more than one dose was required, dantrolene was most often prescribed at intervals of 6 to 8 h. Among possible triggering drugs prescribed within 10 days prior to receiving dantrolene, antipsychotics were prescribed in 169 patients (55.59%) and antidepressants in 134 patients (44.08%) (Table 3). Monoamine oxidase inhibitors were prescribed in 9 (2.96%) cases, succinylcholine in 4 (1.32%), and volatile anesthetic gases in 11 (3.62%).

**Table 3:** Drugs prescribed 10 days before and up to the first date of dantrolene administration (*N* = 304)

Drug Class	N	(%)
**Antipsychotics**	**169**	**(55.59)**
**Antidepressants**	**134**	**(44.08)**
**Dopaminergic Drugs**	**46**	**(15.13)**
**Volatile Anesthetic Gases**	**11**	**(3.62)**
**Monoamine Oxidase Inhibitors**	**9**	**(2.96)**
**Succinylcholine**	**4**	**(1.32)**
**Triptans**	**1**	**(0.33)**

Regarding clinical characteristics, vital signs in patients prior to receiving dantrolene ranged widely overlapping with the normal range (Table 4), but mean maximum values of temperature, heart rate, respiratory rate and blood pressure were elevated. Laboratory values prior to dantrolene were within normal limits on average with wide ranges (Table 4), but mean minimum values of pO_2_ and pulse oximetry as well as maximum serum creatine kinase (CK) levels suggested hypoxia and rhabdomyolysis were common findings. Patients with more extreme values are at high risk for complications and death, and may be more likely to receive and perhaps benefit from dantrolene treatment. This is especially true for the patients with extreme temperature elevations as 55 (18.1%) had temperatures ≥ 40 °C reaching the extreme of 42.4 °C, which constitute a medical emergency risking brain damage or death if not rapidly reduced. Similarly, extreme CK elevations reaching a maximum of 78,600 IU represent massive rhabdomyolysis at risk for myoglobinuric renal failure. Severe hypoxia evidenced by low pO_2_ indicate possible respiratory failure although pO_2_ values may be skewed by supplemental oxygenation and venous blood sampling.

**Table 4:** Vital signs and laboratory values 10 days before and up to the first date of dantrolene administration (*N* = 304)

Measure	N	Mean^a^	Median^a^	SD^b^	Minimum	Maximum	25^th^ percentile	75^th^ percentile
**Temperature (°C)**	**289**	**38.7**	**38.6**	**1.3**	**34.9**	**42.4**	**37.7**	**39.6**
**Pulse (bpm)**	**289**	**116.33**	**117**	**22.80**	**67**	**192**	**100**	**130**
**Respiration (/min)**	**289**	**27.75**	**25**	**9.58**	**13**	**94**	**22**	**32**
**Systolic BP (mmHg)**	**289**	**160.80**	**160**	**26.57**	**69**	**272**	**143**	**174**
**Diastolic BP (mmHg)**	**289**	**93.09**	**94**	**16.98**	**37**	**176**	**82**	**101**
**Pulse oximetry (%)**	**247**	**91.69**	**92**	**5.34**	**62**	**99**	**97**	**99**
**Potassium (mEq/L)**	**301**	**4.69**	**4.50**	**0.84**	**3.20**	**8.22**	**4.10**	**5.00**
**Bicarbonate (mEq/L)**	**192**	**25.31**	**25.40**	**5.06**	**13.3**	**53.3**	**22.7**	**28.2**
**Serum CK**^**c**^ **(IU)**	**279**	**2,859.37**	**860.00**	**6,646.88**	**13**	**78,600**	**226**	**2,411**
**pH**	**191**	**7.34**	**7.38**	**0.13**	**6.95**	**7.59**	**7.26**	**7.43**
**pO**_**2**_^**d**^ **(mmHg)**	**159**	**74.93**	**65.80**	**40.16**	**15.00**	**278.60**	**49.70**	**84.00**
**pCO**_**2**_^**e**^ **(mmHg)**	**197**	**45.57**	**41.90**	**15.40**	**17.00**	**115.00**	**35.50**	**51.5**

^a^mean/median maximum values (mean/median minimum values for pulse oximetry, pO_2_ and pH)

^b^*SD* standard deviation

^c^*CK* creatine kinase

^d^pO_2_ partial pressure of oxygen (pO2 data is likely skewed by the use of supplemental oxygen and venous blood gas sampling)

^e^pCO_2_ partial pressure of carbon dioxide

Overall, 73/304 (24.01%) patients receiving dantrolene died during the index admission. Death occurred in 1/13 (7.69%) patients with MH, 22/108 (20.37%) patients with NMS, and 20/47 (42.55%) patients with severe sepsis. Among patients who *did not* receive dantrolene in the database as a whole, there was one death among 19 (5.26%) patients with the diagnosis of MH, 43/646 (6.66%) deaths among patients with NMS, and 22,233/53,050 (41.91%) deaths among patients with severe sepsis. Although no patients with heatstroke received dantrolene, there were 1,283 cases of heatstroke not treated with dantrolene in the database, with 6 (0.47%) reported deaths. The most frequent complications arising during hospitalization were respiratory (*N* = 65; 21.38%), renal (*N* = 49; 16.12%) or cardiac failure (*N* = 21; 6.91%), and rhabdomyolysis (*N* = 21; 6.91%). The mean overall length of stay for the entire sample was 37.84 ± 81.31 days, with lengths of stay in intensive care of 10.76 ± 21.04 days (medical; *N* = 261) and 10.51 ± 12.15 days (surgical; *N* = 61).

## Discussion

This study found that intravenous dantrolene was administered in 304 hospitalized veterans nationwide over a 10-year period at a rate of about 30 cases per year. On average, patients received a total of about 765 mg of dantrolene over two and a half days. Our primary hypothesis was confirmed that in over 95% of cases, dantrolene was prescribed for diagnoses other than MH. Although the lifesaving benefit of having dantrolene immediately available for the treatment of MH in operative settings is well-established [46], the cost-effectiveness of dantrolene for other syndromes with similar hypermetabolic symptoms remains uncertain. The theoretical rationale for the use of dantrolene in these syndromes other than MH derives by analogy from its proposed mechanism of action in MH; namely, that regardless of the various triggers that may cause symptoms, e.g., antipsychotics in NMS, ambient heat in heatstroke, or inflammatory response in sepsis, dantrolene may reduce hypermetabolic activity in muscle and the resulting systemic symptoms by sequestration of intracellular calcium in the sarcoplasmic reticulum.

Data were also extracted to determine whether a targeted symptom profile prompting dantrolene treatment could be identified apart from a formal diagnosis of MH. To some extent, the demographics of dantrolene-treated patients are nonspecific and reflect the population served by the VA (i.e., predominately older males with chronic medical co-morbidities). However, patients with underlying psychiatric and substance use disorders receiving psychotropic medications were overrepresented. Among dantrolene-treated patients, over one third were diagnosed with NMS during hospitalization, one half had a psychiatric diagnosis, one quarter had substance use diagnoses, and at least half received either antipsychotics or antidepressants. While the majority were initially admitted to medical services, nearly all received dantrolene there except for 14% who were on surgical services when dantrolene was administered. Reasons for differences in geographic distributions of the sample across regions in the US are unknown but higher rates of dantrolene use in the Midwest and Continental states could represent different prescribing practices using triggering drugs or dantrolene, different demographics of veterans in each region, or possible concentration of family or ethnic groups with genetic MH-susceptibility in certain regions.

Clinicians prescribed dantrolene for patients who on average showed elevated temperatures, tachycardia, tachypnea, and hypertension. Laboratory values varied widely, but patients often presented with hypoxia and elevated CK levels. MH-triggering drugs succinylcholine or volatile anesthetics were prescribed in a fraction of cases. The most common drugs prescribed prior to dantrolene treatment were antipsychotics and antidepressants. Even though one third of patients receiving dantrolene were diagnosed with NMS, the presence of antipsychotics in 55.59% is unexplained. Similarly, while antidepressants are associated with serotonin syndrome and were prescribed in nearly half of the patients, only 3.29% were diagnosed with possible serotonin syndrome. Reasons for over-representation of psychotropic drugs are unclear, but may suggest an increased risk of hypermetabolic crises in patients with psychiatric or substance use diagnoses who receive antipsychotics or antidepressants, or clinicians are inclined to implicate drug-related etiologies for elevated temperatures when these drugs are present. Alternatively, these findings of psychiatric co-morbidities and psychotropic treatment may simply represent an artefact of the veteran population. Monoamine oxidase inhibitors, which are associated with severe serotonergic toxicity, were prescribed in less than 5% of cases. Although anti-migrainous triptans have been implicated in serotonin syndrome, only one patient received triptans in the study. Statin drugs have been associated with rhabdomyolysis and myopathy [31], but no patients in our sample were diagnosed with underlying myopathies.

The gravity of the critically-ill patients for whom clinicians prescribe dantrolene is evidenced by the high overall mortality rate of 24.01%. Dantrolene, the drug of choice for MH which was frequently fatal in the past [3, 6, 7], should be available wherever triggering anesthetics are utilized [7]. This includes succinylcholine which was the only triggering drug administered in two of the 13 patients diagnosed with MH who received dantrolene. Only one death occurred among 13 patients (1/13; 7.69%) diagnosed with MH who received dantrolene. However, only 13/32 (40.63%) patients diagnosed with MH in the database were treated with dantrolene and only one death was also reported among the 19 patients (5.26%) who did *not *receive dantrolene. The apparently similar mortality with or without dantrolene may be explained by the likelihood that mortality was reduced by dantrolene in patients who met criteria for MH, whereas mortality was also low in patients for whom dantrolene was not prescribed because their symptoms were not severe enough to meet threshold criteria for MH and warrant dantrolene treatment [7], or because their MH-like episodes were misdiagnosed and actually were caused by other syndromes treated with alternative measures. A previous study suggested that only one fourth of cases recorded as MH in hospital records represented true MH cases, whereas an equal number were attributed to other conditions [8, 9]. In our study, a secondary subgroup inspection of individual medical records of MH patients who received dantrolene revealed that 7/13 (53.8%) were “somewhat greater than likely” to meet standardized criteria for a definite MH episode [44], compared with only 1/19 (5.26%) patients coded as having MH who did *not* receive dantrolene. The one patient who did not receive dantrolene but was rated as somewhat greater than likely to have MH after general anesthesia developed only mild symptoms that were caught quickly and resolved before dantrolene could be administered. Another patient who did not receive dantrolene and died was incorrectly coded as having MH but actually died from extreme hyperthermia in the context of status epilepticus unrelated to anesthesia. The Malignant Hyperthermia Association of the United States offers web-based information on managing an MH crisis (www.mhaus.org) and a toll-free hotline (1–800-644–9737), which could provide consultation on the differential diagnosis of MH and indications for dantrolene, although the hotline was contacted in only six patients with MH in the survey.

In the sample, NMS was diagnosed in 35.55% of the patients who received dantrolene, similar to two previous samples [42, 47]. Although previous reports documented improved survival of NMS following dantrolene treatment [34–37, 42, 47], not all have been favorable [39, 41, 48]. Recent guidelines recommend specific treatments based on character, duration and severity NMS symptoms, with dantrolene reserved for patients showing extreme temperature elevations and severe symptoms [49]. Perhaps for this reason, only 108 (14.32%) of 754 patients diagnosed with NMS in the database received dantrolene. Dantrolene was likely used as a last resort in a minority of patients with poor prognoses and significant co-morbidities or severe symptoms. Although the fact that treated patients showed higher mortality compared with NMS cases not receiving dantrolene suggests it may be ineffective or worse, the effectiveness of dantrolene in NMS cannot be addressed by our data because groups were neither randomized, controlled, blinded, nor matched [42]. For example, concurrent sepsis or other comorbidities and symptom severity have been identified as significant predictors of mortality and response in NMS in previous studies [38, 42, 48, 50, 51]. Evidence on the efficacy of dantrolene for NMS is inconclusive pending studies using standardized diagnostic criteria that also control for co-morbidities and severity of symptoms [52–54].

The effectiveness of administering dantrolene in patients diagnosed with other disorders is similarly uncertain. Despite previous evidence suggesting its therapeutic value [29], dantrolene was prescribed in less than 1% of patients with severe sepsis in our study with uniformly dire outcomes regardless of treatment. Although evidence suggests MH-susceptibility may be a risk factor for exertional heatstroke [20–22, 47], no cases of heatstroke were treated with dantrolene in the VA sample and the low mortality rate suggests it was effectively treated with supportive measures. It was unclear whether rhabdomyolysis was recorded as an indication for dantrolene or a complication of the syndromes being treated. Patients with Parkinson’s disease may experience hypermetabolic reactions after discontinuation of dopamine therapies, which may contribute to the fact that 7.24% of patients were diagnosed with Parkinson’s disease in the study. Symptoms can be reversed with reinstitution of dopaminergic agents in such cases [28]. Serotonin toxicity, which is treated by drug withdrawal, supportive care and serotonin antagonists, was reported in few cases. Possible masseter muscle rigidity and stimulant abuse were implicated in single cases receiving dantrolene. There were no cases of diabetes with hyperosmolarity, or cases with other underlying myopathies.

The strength of the study includes use of a large clinical database. However, retrospective data depend on precision in diagnosis and coding of records. Diagnoses were based on clinician judgment without standardized criteria or confirmatory tests [44, 52, 53]. ICD-9-CM codes are often approximate. Diagnoses were not mutually exclusive such that patients could have one or more diagnoses implicated in hypermetabolic syndromes. In addition, while we focused on diagnoses of hypermetabolic syndromes recorded during hospital admissions, we cannot be sure that these diagnoses per se specifically prompted administration of dantrolene in these patients. The list of syndromes we selected to search for dantrolene treatment was not exhaustive such that other hypermetabolic syndromes, e.g., thyroid storm, pheochromocytoma, that may be treated with dantrolene may have been missed. The 10 day interval prior to dantrolene initiation for potential triggering drugs may be too great and possibly captured ongoing maintenance treatment with drugs unrelated to the episode. However, there is a wide variation in onset of symptoms after exposure; MH may occur within minutes to hours whereas two-thirds of NMS episodes occur within one to two weeks. We did not extract data on polypharmacy and therefore cannot exclude drug interactions as a factor, with patients commonly receiving more than one agent capable of inducing hypermetabolic symptoms. Descriptive clinical signs elicited on physical examination such as muscle rigidity that are recorded in narrative fashion in progress notes were not available. As seen in Table 4, vital signs and laboratory data are not distributed normally and may be skewed by more severely affected patients with extreme outlier values, while pO2 values in particular may be confounded by supplemental oxygen use and venous blood sampling. In addition, vital signs and laboratory values were defined as the maximum value within the 10 days before dantrolene was administered to compose a standardized profile of the most severe point of the syndrome; a categorical description of values simply as abnormal was not possible because the database did not record whether any specific values that were defined as abnormal per se led to dantrolene administration. Finally, not all values were available in the database for all patients in the sample (Table 4). We cannot be sure whether these data were not obtained or were obtained but not recorded correctly in the database, and to what extent missing data may have influenced the decision to prescribe dantrolene and the results of our survey. While we selected acute and common complications that have been associated with MH in hospital, other complications of hyperthermia may occur and are important to consider, especially possible effects on brain function, e.g., cognitive defects, movement disorders, that could be persistent. Finally, the generalizability of the results may be limited to veterans receiving care within the VA system, a homogeneous population compared to community samples.

## Conclusions

In conclusion, intravenous dantrolene was administered to 304 patients in the VA healthcare system nationwide over a 10-year period with a variety of disorders characterized by a range of elevated temperatures, CK levels, and associated hypermetabolic symptoms. MH accounted for only 4.28% of cases receiving dantrolene, with NMS (35.53%) and sepsis (15.46%) as the most frequent diagnoses recorded during hospitalizations associated with dantrolene administration. Although dantrolene was administered with a relatively favorable outcome in patients diagnosed with definite MH, the only approved indication for the drug, uncontrolled exploratory survey data suggested that dantrolene appeared to have no significant effect on mortality in sepsis and was associated with increased mortality in NMS compared to patients who did not receive this treatment. However, the effectiveness and safety of dantrolene treatment for hypermetabolic syndromes other than MH remains uncertain in the absence of controlled studies that include groups with diagnoses confirmed by standardized criteria and matched for co-morbidities and symptom severity in these critically-ill patients.

## Supplementary Information

**Additional file 1: Supplementary Table 1.** Diagnostic Codes (International Classification of Diseases, Ninth Revision, Clinical Modification [ICD-9-CM]).

## Data Availability

A de-identified, anonymized study-specific dataset excluding names and 38 USC §7332-protected information, will be shared, pursuant to a written request and IRB approved waiver of HIPAA authorization, with the approval of the Under Secretary for Health, in accordance with VHA Handbook 1605.1 §13.b(1)(b) or §13.b(1)(c) or superseding versions of that Handbook, and a written assurance from the recipient that; the information will be maintained in accordance with the security requirements of 38 CFR Part 1.466, or more stringent requirements; the information will not be re-disclosed except back to VA; and the information will not identify any individual patient in any report of the research or otherwise disclose patient identities. The corresponding author (SC) should be contacted for further information to request the data from this study.

## References

[1] Halsall PJ, Ellis FR (1983). The control of muscle contracture by the action of dantrolene on the sarcolemma. Acta Anaesthesiol Scand.

[2] Kolb ME, Horne ML, Martz R (1982). Dantrolene in human malignant hyperthermia. Anesthesiology.

[3] Rosenberg H, Pollock N, Schiemann A, Bulger T, Stowell K (2015). Malignant hyperthermia: a review. Orphanet J Rare Dis.

[4] Biesecker LG, Dirksen RT, Girard T (2020). Genomic screening for malignant hyperthermia susceptibility. Anesthesiology.

[5] Brandom BW, Larach MG, Chen MS, Young MC (2011). Complications associated with the administration of dantrolene 1987 to 2006: a report from the North American Malignant Hyperthermia Registry of the Malignant Hyperthermia Association of the United States. Anesth Analg.

[6] Riazi S, Kraeva N, Hopkins PM (2018). Updated guide for the management of malignant hyperthermia. Can J Anaesth.

[7] Larach MG, Gronert GA, Allen GC, Brandom BW, Lehman EB (2010). Clinical presentation, treatment, and complications of malignant hyperthermia in North America from 1987 to 2006. Anesth Analg.

[8] Lu Z, Rosenberg H, Brady JE, Li G (2016). Prevalence of malignant hyperthermia diagnosis in New York State Ambulatory Surgery Center Discharge Records 2002 to 2011. Anesth Analg.

[9] Pinyavat T, Rosenberg H, Lang BH (2015). Accuracy of malignant hyperthermia diagnoses in hospital discharge records. Anesthesiology.

[10] Gonsalves SG, Ng D, Johnston JJ (2013). Using exome data to identify malignant hyperthermia susceptibility mutations. Anesthesiology.

[11] Zhao X, Song Q, Gao Y (2014). Hypothesis: exertional heat stroke-induced myopathy and genetically inherited malignant hyperthermia represent the same disorder, the human stress syndrome. Cell Biochem Biophys.

[12] Snoeck M, Treves S, Molenaar JP, Kamsteeg EJ, Jungbluth H, Voermans NC (2016). "Human stress syndrome" and the expanding spectrum of RYR1-related myopathies. Cell Biochem Biophys.

[13] Kruijt N, den Bersselaar LV, Snoeck M (2022). RYR1-related rhabdomyolysis: a spectrum of hypermetabolic states due to ryanodine receptor dysfunction. Curr Pharm Des.

[14] Caroff SN, Watson CB, Rosenberg H (2021). Drug-induced hyperthermic syndromes in psychiatry. Clin Psychopharmacol Neurosci.

[15] Tobin JR, Jason DR, Challa VR, Nelson TE, Sambuughin N (2001). Malignant hyperthermia and apparent heat stroke. JAMA.

[16] Brandom BW, Muldoon SM (2013). Unexpected MH deaths without exposure to inhalation anesthetics in pediatric patients. Paediatr Anaesth.

[17] Gronert GA, Thompson RL, Onofrio BM (1980). Human malignant hyperthermia: awake episodes and correction by dantrolene. Anesth Analg.

[18] Kraeva N, Sapa A, Dowling JJ, Riazi S (2017). Malignant hyperthermia susceptibility in patients with exertional rhabdomyolysis: a retrospective cohort study and updated systematic review. Can J Anaesth.

[19] Capacchione JF, Muldoon SM (2009). The relationship between exertional heat illness, exertional rhabdomyolysis, and malignant hyperthermia. Anesth Analg.

[20] Fiszer D, Shaw MA, Fisher NA (2015). Next-generation Sequencing of RYR1 and CACNA1S in malignant hyperthermia and exertional heat illness. Anesthesiology.

[21] Laitano O, Murray KO, Leon LR (2020). Overlapping mechanisms of exertional heat stroke and malignant hyperthermia: evidence vs. conjecture. Sports Med.

[22] Hadad E, Cohen-Sivan Y, Heled Y, Epstein Y (2005). Clinical review: Treatment of heat stroke: should dantrolene be considered?. Crit Care.

[23] Caroff SN, Mann SC (1993). Neuroleptic malignant syndrome. Med Clin North Am.

[24] Levenson JL (1985). Neuroleptic malignant syndrome. Am J Psychiatry.

[25] Gerbershagen MU, Wappler F, Fiege M (2003). Effects of a 5HT(2) receptor agonist on anaesthetized pigs susceptible to malignant hyperthermia. Br J Anaesth.

[26] Russell T, Riazi S, Kraeva N, Steel AC, Hawryluck LA (2012). Ecstacy-induced delayed rhabdomyolysis and neuroleptic malignant syndrome in a patient with a novel variant in the ryanodine receptor type 1 gene. Anaesthesia.

[27] Grunau BE, Wiens MO, Brubacher JR (2010). Dantrolene in the treatment of MDMA-related hyperpyrexia: a systematic review. CJEM.

[28] Newman EJ, Grosset DG, Kennedy PG (2009). The parkinsonism-hyperpyrexia syndrome. Neurocrit Care.

[29] Wei H, Liang G, Vera RM (2021). Dantrolene repurposed to treat sepsis or septic shock and COVID-19 patients. Eur Rev Med Pharmacol Sci.

[30] Siegmueller C, Narasimhaiah R (2010). Fatal 2,4-dinitrophenol poisoning... coming to a hospital near you. Emerg Med J.

[31] Vladutiu GD, Isackson PJ, Kaufman K (2011). Genetic risk for malignant hyperthermia in non-anesthesia-induced myopathies. Mol Genet Metab.

[32] Zeitler P, Haqq A, Rosenbloom A, Glaser N, rugs, Therapeutics Committee of the Lawson Wilkins Pediatric Endocrine S (2011). Hyperglycemic hyperosmolar syndrome in children: pathophysiological considerations and suggested guidelines for treatment. J Pediatr.

[33] Coffey RJ, Edgar TS, Francisco GE (2002). Abrupt withdrawal from intrathecal baclofen: recognition and management of a potentially life-threatening syndrome. Arch Phys Med Rehabil.

[34] Sakkas P, Davis JM, Janicak PG, Wang ZY (1991). Drug treatment of the neuroleptic malignant syndrome. Psychopharmacol Bull.

[35] Rosenberg MR, Green M (1989). Neuroleptic malignant syndrome. Review of response to therapy. Arch Intern Med.

[36] Tsutsumi Y, Yamamoto K, Matsuura S, Hata S, Sakai M, Shirakura K (1998). The treatment of neuroleptic malignant syndrome using dantrolene sodium. Psychiatry Clin Neurosci.

[37] Yamawaki Y, Morio M, Kazamutsuri G (1993). Clinical evaluation and effective usage of dantrolene sodium in neuroleptic malignant syndrome. Kiso to Rinsyou (Clinical Reports).

[38] Kuhlwilm L, Schönfeldt-Lecuona C, Gahr M, Connemann BJ, Keller F, Sartorius A (2020). The neuroleptic malignant syndrome-a systematic case series analysis focusing on therapy regimes and outcome. Acta Psychiatr Scand.

[39] Rosebush P, Stewart T (1989). A prospective analysis of 24 episodes of neuroleptic malignant syndrome. Am J Psychiatry.

[40] Ngo V, Guerrero A, Lanum D (2019). Emergent treatment of neuroleptic malignant syndrome induced by antipsychotic monotherapy using dantrolene. Clin Pract Cases Emerg Med.

[41] Reulbach U, Dutsch C, Biermann T (2007). Managing an effective treatment for neuroleptic malignant syndrome. Crit Care.

[42] Pawar SC, Rosenberg H, Adamson R, LaRosa JA, Chamberlain R (2015). Dantrolene in the treatment of refractory hyperthermic conditions in critical care: a multicenter retrospective study. Open J Anesthesiol.

[43] International Classification of Diseases,Ninth Revision, Clinical Modification (ICD-9-CM). https://www.cdc.gov/nchs/icd/icd9cm.htm. Accessed 19 Jan 2022.

[44] Larach MG, Localio AR, Allen GC (1994). A clinical grading scale to predict malignant hyperthermia susceptibility. Anesthesiology.

[45] Malignant Hyperthermia Association of the United States: Managing a crisis. https://www.mhaus.org/healthcare-professionals/managing-a-crisis/. Accessed 19 Jan 2022.

[46] Aderibigbe T, Lang BH, Rosenberg H, Chen Q, Li G (2014). Cost-effectiveness analysis of stocking dantrolene in ambulatory surgery centers for the treatment of malignant hyperthermia. Anesthesiology.

[47] Doucet A, Langlois H, Villeneuve E (2019). Efficacy and safety of dantrolene in toxic hyperthermia: a systematic review.

[48] Nakamura M, Yasunaga H, Miyata H, Shimada T, Horiguchi H, Matsuda S (2012). Mortality of neuroleptic malignant syndrome induced by typical and atypical antipsychotic drugs: a propensity-matched analysis from the Japanese Diagnosis Procedure Combination database. The J Clin Psychiatry.

[49] Strawn JR, Keck PE, Caroff SN (2007). Neuroleptic malignant syndrome. Am J Psychiatry.

[50] Modi S, Dharaiya D, Schultz L, Varelas P (2016). Neuroleptic malignant syndrome: complications, outcomes, and mortality. Neurocrit Care.

[51] Guinart D, Misawa F, Rubio JM (2021). A systematic review and pooled, patient-level analysis of predictors of mortality in neuroleptic malignant syndrome. Acta Psychiatr Scand.

[52] Yacoub A, Francis A (2006). Neuroleptic malignant syndrome induced by atypical neuroleptics and responsive to lorazepam. Neuropsychiatr Dis Treat.

[53] Sachdev PS (2005). A rating scale for neuroleptic malignant syndrome. Psychiatry Res.

[54] Gurrera RJ, Caroff SN, Cohen A (2011). An international consensus study of neuroleptic malignant syndrome diagnostic criteria using the Delphi method. J Clin Psychiatry.

