# Supplementary material for: Intravenous dantrolene in hypermetabolic syndromes: a survey of the U.S. Veterans Health Administration database
Source: BMC Anesthesiol. 2022 Sep 19;22:298. doi: 10.1186/s12871-022-01841-z (PMC9484236; doi:10.1186/s12871-022-01841-z)
Supplement: Supplementary file 1 — Additional file 1: Supplementary Table 1. Diagnostic Codes (International Classification of Diseases, Ninth Revision, Clinical Modification [ICD-9-CM]). [file 12871_2022_1841_MOESM1_ESM.docx]

**Supplementary Table 1. Diagnostic Codes (International Classification of Diseases, Ninth Revision, Clinical Modification [ICD-9-CM])**

**Diagnostic codes associated with dantrolene treatment**

Neuroleptic Malignant Syndrome 333.92

Severe Sepsis 785.52 or 995.92

Rhabdomyolysis 728.88

Fever of unknown origin 780.60

Parkinson’s disease 332.0

Malignant hyperthermia 995.86

Serotonin syndrome 333.99

Masseter muscle rigidity 728.85

Stimulant use 304.4x or 305.7x

Heatstroke 992.0

Myopathies 359.x

Diabetes with hyperosmolarity 250.20

**Diagnostic codes for possible complications arising during hospitalization**

Acute renal failure 584.9

Rhabdomyolysis 728.88

Disseminated intravascular coagulation 286.6,

Pulmonary embolus 415.1

Cardiac arrest 427.5

Congestive heart failure 428.0

Respiratory failure 518.xx

Phlebitis 451.89

Compartment syndrome 958.90
